# A Small Fraction of Strongly Cooperative Sodium Channels Boosts Neuronal Encoding of High Frequencies

**DOI:** 10.1371/journal.pone.0037629

**Published:** 2012-05-29

**Authors:** Min Huang, Maxim Volgushev, Fred Wolf

**Affiliations:** 1 Max-Planck-Institute for Dynamics and Self-Organization, Bernstein Center for Computational Neuroscience, and Faculty of Physics, Georg August University School of Science (GAUSS), Göttingen, Germany; 2 State Key Laboratory of Cognitive Neuroscience and Learning, Beijing Normal University, Beijing, China; 3 Department of Psychology, University of Connecticut, Storrs, Connecticut, United States of America; University of Houston, United States of America

## Abstract

Generation of action potentials (APs) is a crucial step in neuronal information processing. Existing biophysical models for AP generation almost universally assume that individual voltage-gated sodium channels operate statistically independently, and their avalanche-like opening that underlies AP generation is coordinated only through the transmembrane potential. However, biological ion channels of various types can exhibit strongly cooperative gating when clustered. Cooperative gating of sodium channels has been suggested to explain rapid onset dynamics and large threshold variability of APs in cortical neurons. It remains however unknown whether these characteristic properties of cortical APs can be reproduced if only a fraction of channels express cooperativity, and whether the presence of cooperative channels has an impact on encoding properties of neuronal populations. To address these questions we have constructed a conductance-based neuron model in which we continuously varied the size of a fraction 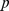 of sodium channels expressing cooperativity and the strength of coupling between cooperative channels 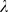. We show that starting at a critical value of the coupling strength 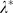, the activation curve of sodium channels develops a discontinuity at which opening of all coupled channels becomes an all-or-none event, leading to very rapid AP onsets. Models with a small fraction, 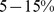, of strongly cooperative channels generate APs with the most rapid onset dynamics. In this regime APs are triggered by simultaneous opening of the cooperative channel fraction and exhibit a pronounced biphasic waveform often observed in cortical neurons. We further show that presence of a small fraction of cooperative Na+ channels significantly improves the ability of neuronal populations to phase-lock their firing to high frequency input fluctuation. We conclude that presence of a small fraction of strongly coupled sodium channels can explain characteristic features of cortical APs and has a functional impact of enhancing the spike encoding of rapidly varying signals.

## Introduction

**Figure 1:**
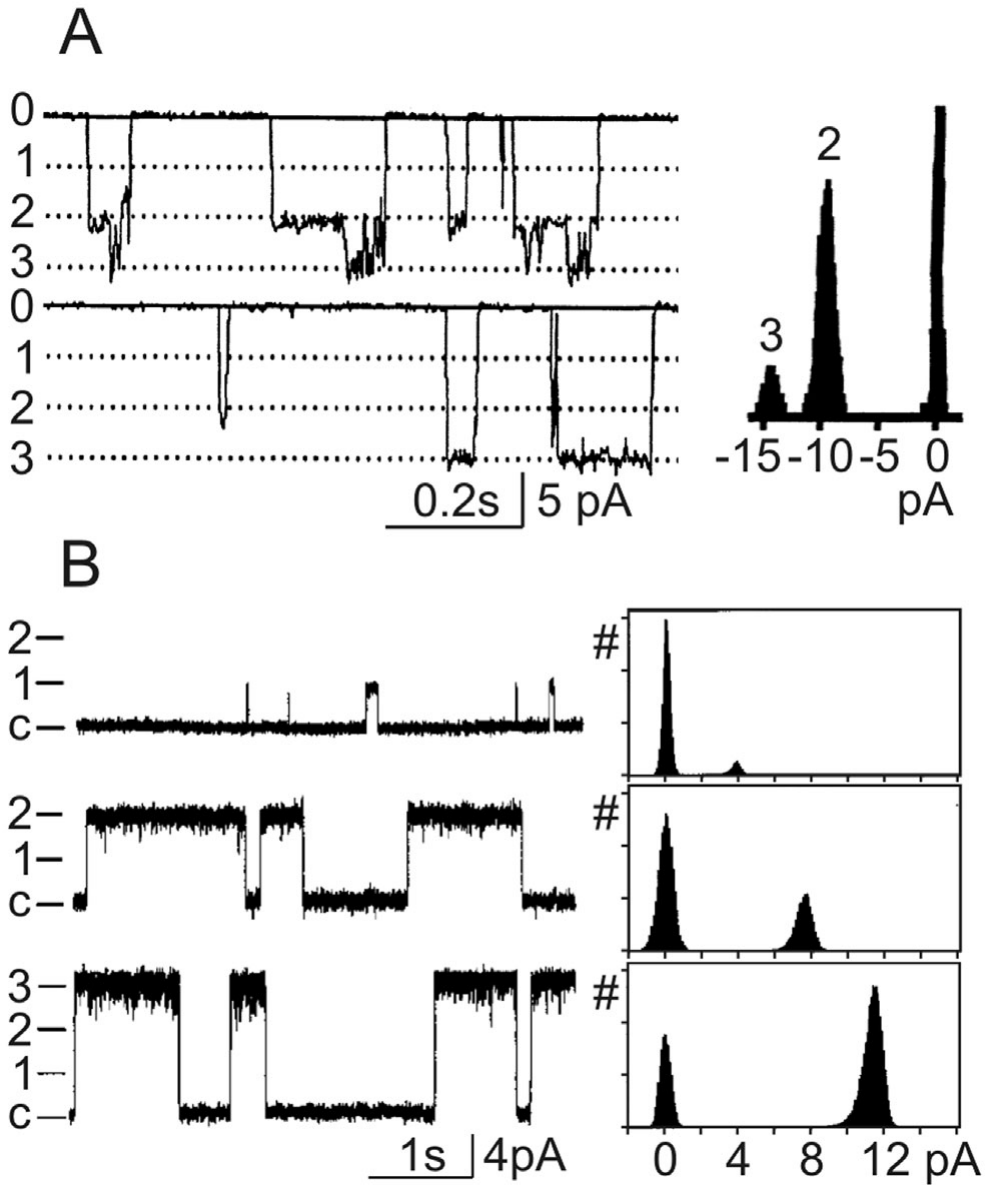
Cooperative gating of 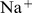 and 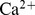 channels. (**A**) Simultaneous openings of pairs and triples of 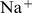 channels in inside-out patch from cardiac myocytes treated with the ischaemic metabolite lysophosphatidylchloline [2]. In the left panel, zero corresponds to closed state; dotted lines and numbers 1,2,3 indicate openings to single, double and triple unitary conductance levels. Right panel shows histogram of current amplitude distribution. Note frequent occurrence of openings to double and triple unitary levels, but no openings to the unitary level. (**B**) Coopled gating of ryanodine R2 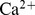 channels in cardiac cells [6]. Left panel shows example traces with openings to single, double and triple unitary conductance levels. Closed state is indicated by c; single, double and triple unitary conductance levels are indicated by 1,2,3. Right panel shows current amplitude histograms, corresponding to the traces on the left. Reproduced with permision from [2] and [6].

Ion channels are integral membrane proteins which, depending on conformation, can pass ionic currents and thus induce dynamic changes in membrane potential [1]. In voltage gated channels, permeability for ions is controlled by the membrane potential, introducing a fundamental nonlinearity in electrical signaling in neurons and muscle cells. An avalanche-like opening of voltage gated channels produces in these cells pulse-like electrical signals, action potentials (APs), which underlie the information processing capabilities of neurons. Biophysical models for AP generation almost universally assume that individual channels open and close statistically independently and are coupled only through the transmembrane voltage. However, channels for physiologically important cations (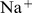, 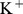, 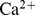) have been found capable of cooperative gating when clustered [2]–[8]. Fig. 1 shows examples of coupled gating of sodium and calcium channels in cardiac myocytes. Sodium channels express coupled gating after treatment with the ischaemic metabolite lysophosphatidylchloline [2] (Fig. 1A). Coupled gating of pairs and triplets of channels was reported for ryanodin R2 channels that lead to release of calcium from sarcoplasmic reticulum in cardiac cells [6] (Fig. 1B). In both examples, transitions between zero and conductance levels corresponding to opening of 2–3 channels occur more frequently than transitions to single-channel conductance level, indicating coupled gating of 2–3 channels. For potassium channels, coupled gating of up to 5 channels has been reported [3].

Cooperative gating of ion channels has been proposed to represent a general capability of proteins to undergo conformational spread [9]. It coordinates the gating of individual channels, such that the opening of one channel increases the probability of opening of neighboring channels. Examples of channels exhibiting cooperative gating include 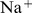 channels [2], 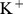 channels [3], 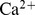 channels [5], [6] and ligand-gated receptors [7], [8]. Cooperative gating of 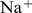 channels has been hypothesized to underlie the observed rapid onset dynamics of APs in cortical neurons [10]. An alternative hypothesis attributes the rapid AP onset to lateral currents within the neuron [11].

Prior theoretical analysis has demonstrated that the onset dynamics of AP generators critically affects coding abilities of spiking neurons [12]–[19]. If spike encoding is determined by AP onset dynamics at the site of its initiation, an assessment of coding properties might help to distinguish between the competing hypotheses [10], [20]. However, it is currently not known how strong and how prevalent channel cooperativity has to be, in order to have a sizable impact on the AP onset dynamics and neural coding properties. Here we set out to address these questions using a conductance based neuron model in which (i) a fraction 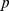 of 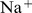 channels exhibiting cooperative gating and (ii) the strength of inter-channel coupling 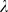 can be continuously varied.

## Methods

### Model of Cooperative Gating of Sodium Channels

Cooperative gating of 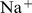 channels was modelled as following. We assume that a channel is coupled to 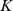 neighboring channels such that the opening of each neighbor increases the probability of the channel to open. Kinetics of an activation variable 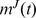 is then described by:
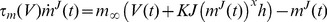
(1)Here 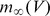 is the steady state activation curve of an isolated channel or a population of independent channels, 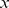 is the exponent of the activation function, 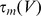 is the activation time constant, 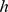 is the fraction of channels available for activation. The probability that a channel is in open state is then 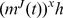, hence the expected number of open neighbor channels that are coupled to the channel is 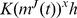. 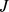 is a coupling constant. It has units of mV, and measures the strength of coupling by the shift along the activation curve that would increase the open probability of an isolated channel by the same amount as opening of a coupled channel. Note that operationally, a right-hand shift along the activation curve is equivalent to the left-hand shift of the curve itself. Coupling strength depends on both, the coupling constant 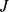 and the number of coupled neighboring channels 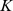. Eq. 1 represents the mean field approximation of a coupled population of Markov models for the individual channels [10]. For 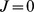, Eq. 1 reduces to the canonical case of independent channel activation.

It is known that inactivation of 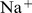 channels depends on the channel state rather than voltage: open channels inactivate fast at any voltage by proceeding from an open state to an absorbing inactivation state [1], [21]–[23].

To implement this we made the kinetics of inactivation for the cooperative channel fraction dependent on the fraction of open channels. The simplest choice for this is 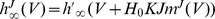 and 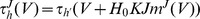 which increases the rate of inactivation when the cooperative fraction opens. Note that this does not mean that the process of inactivation is itself modelled to be cooperative. Rather, inactivation happens independently but with a rate that increases when the cooperative fraction opens.

We use this formalism to assess signatures of cooperativity in the activation of sodium channels, AP onset dynamics and encoding properties.

### Model with a Fraction of Cooperative Channels

To study the impact of channel cooperativity on AP onset dynamics, waveform shape and coding properties of neuronal populations we constructed a conductance based neuron model in which (i) a fraction 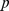 of 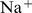 channels exhibiting cooperative gating and (ii) the strength of inter-channel coupling 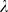 can be continuously varied (see Eq. 7 and related text for definition of 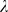). A fraction 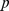 of cooperative channels was included in previously well-characterized Hodgkin-Huxley type neuron model, the Wang-Buzsaki (WB) model [24]. In this cooperative WB model (cWB), the current balance equation reads
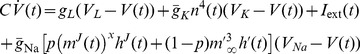
(2)Here, 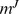 and 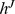 are gating variables for the fraction 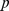 of cooperative sodium channels, 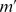 and 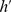 are gating variables for the remaining 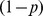 non-cooperative sodium channels. Gating variables and kinetic equations for non-cooperative sodium channels as well as variables and equations for all other channels were as in the WB model [24]. Model code is available as supplement to this paper, File S1.

Encoding properties of populations of neuron models were characterized using the established approaches of accessing frequency response function [12]–[14], [16], [17]. We studied the firing rate dynamics of neuronal populations in response to signals of different frequencies 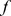 immersed in fluctuating current
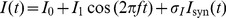
(3)in the model neurons. The constant current level 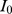 was adjusted to keep the mean firing rate 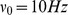. The background synaptic noise 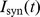 was mimicked by an Ornstein-Uhlenbeck process with correlation time constant 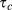. In the linear response regime the instantaneous firing rate of the population fulfills 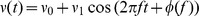. Encoding of input frequency 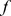 is characterized by the firing rate modulation 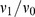 at that frequency. Plotting the firing rate modulation 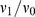 against frequency 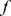 provides the frequency response function of neuronal population.

## Results

### Activation Kinetics of Cooperative Channels

We first assume a Boltzmannian activation curve of a single channel with slope factor 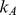


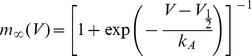
(4)In the limit of very short activation time constant 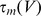, the fraction of open channels 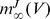 after a step to voltage V, called the collective activation curve is defined by
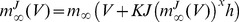
(5)Recent study in central neurons demonstrated that time course of activation of sodium channels was best fitted by a mono-exponential function [25], therefore we start our analysis with 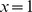. We assessed collective activation curve from responses to voltage steps of increasing amplitude. All voltage steps were applied from the same steady state, so the fraction of available channels is constant, 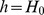.

The steady state solution of 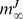 is obtained from the intersection points of the two curves 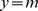 and 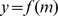, where 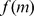 is defined as
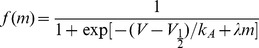
(6)with



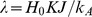
(7)When 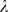 is very small, there is only one intersection point at a small value of 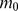 close to 0. When 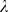 is very large, the intersection point shifts to a value close to 1. In a critical range of 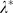, there exist three intersection points at certain ranges of voltages. By increasing 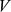, a jump of the intersection point occurs from a small value of the fixed point 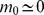 to a larger value near 1. At this transition point, the two curves of 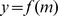 and 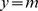 intersect tangentially. The critical value of 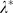 is thus obtained by solving the system
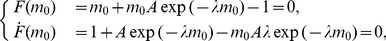
(8)where



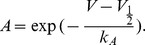



The solutions of 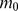 are obtained from
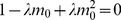
(9)which has real roots only if 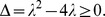 The value of 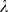 must be positive, hence 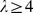. The corresponding coupling strength has to satisfy
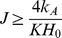
(10)to produce a finite jump in collective activation curve.

**Figure 2:**
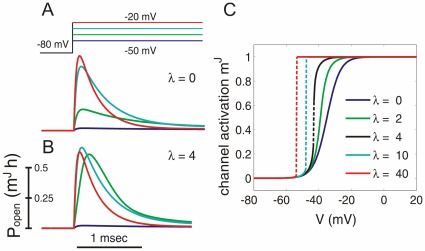
Dependence of the sodium channel dynamics on the coupling strength 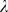 (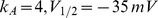). (**A,B**) Simulated open probability of sodium channels in response to steps of holding potential of increasing amplitude. The voltage-clamp protocol is shown above the traces in (**A**). Simulations for independent channels (**A**, 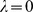) and for cooperative channels with the critical value of coupling strength 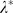 (**B**, 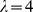). (**C**) Collective activation curves of sodium channels with an increasing coupling strength 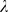. Discontinuous portions of activation curves for 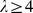 are shown as interrupted lines.

Fig. 2 shows the dependence of the collective activation curve on the effective coupling strength 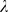 for 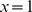. With increasing 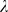, the activation curve first becomes steeper, and when 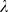 exceeds the critical coupling strength of 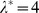 the activation curve develops a discontinuity at a threshold voltage 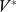.

For stronger coupling (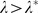) the threshold moves to more negative potentials and the collective activation curve approaches a step function such that almost all channels are closed below 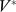 but almost all are open above 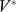. Similar behavior was found for 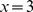, as in original Hodgkin-Huxley model [26] and in numerically simulated voltage clamp experiments using Eq. 1 with sodium channel kinetics as in the Wang-Buzsaki (WB) model [24], 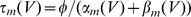 and 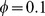 to achieve a peak activation time constant of 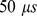 as measured in cortical neurons [25]. In all these simulations, when coupling exceeded the critical value 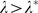, activation of the 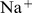 current became basically an all-or-none event.

### Action Potentials in a Model with a Fraction of Cooperative Channels

Next, we assessed whether the presence of only a fraction of cooperative channels is sufficient to achieve rapid AP onset. We used previously well-characterized neuron model of Hodgkin-Huxley type, the Wang-Buzsaki model [24], and included in it a fraction 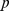 of cooperative 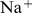 channels. In this cooperative WB (cWB) model, we continuously varied the fraction 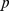 of 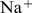 channels exhibiting cooperative gating and the strength of inter-channel coupling 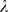.

**Figure 3:**
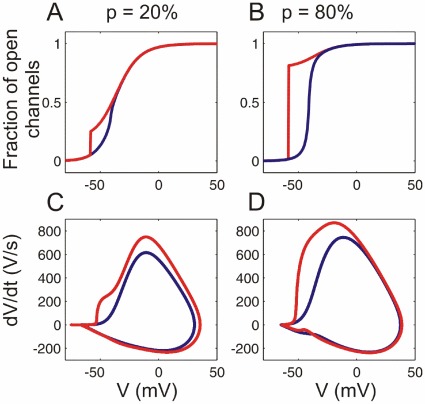
Channel activation and AP dynamics in models with cooperative sodium channels. (**A,B**) Activation curves of sodium channels and (**C.D**) AP phase plots for the models with a small (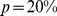) and a large (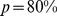) fraction of cooperative channels, and a weak coupling (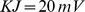, blue traces) or strong coupling (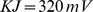, red traces).

In the cooperative WB model, AP waveform and onset dynamics assessed using 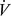 vs. 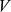 phase plots were very sensitive to the fraction of cooperative channels and the coupling strength (Fig. 3). The AP onset was essentially determined by the activation of the cooperative channel fraction, and its rapidness increased with coupling strength. For a small fraction of strongly cooperative channels the AP waveform was typically biphasic (Fig. 3C). For a large fraction of cooperative channels the AP was monophasic with rapid onset (Fig. 3D). APs were considered biphasic if 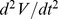 had 3 zero crossings during the AP upstroke, as opposed to 1 zero-crossing for monophasic APs.

**Figure 4:**
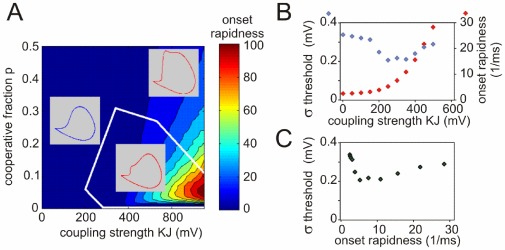
AP onset rapidness and threshold variability in models with cooperative channels. (**A**) Dependence of the AP onset rapidness measured as the phase slope at 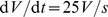 on the coupling strength and the fraction of cooperative channels. White contours delimit the bounder lines between the monophasic and the biphasic APs (see insets). (**B**) Dependence of the threshold variability (blue symbols, scale on the left) and onset rapidness (red symbols, scale on the right) on the coupling strength 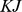 for a model with a small fraction (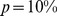) of cooperative channels. Fluctuating current for injection that mimicked background synaptic noise was synthesized as described in Methods (see Eq. 3) (**C**) Relation between threshold variability and AP onset rapidness for the same model, data from (**B**).

This behavior was robust to the details of sodium channel models, *e.g.*, when the kinetics of the cooperative fraction was constructed from 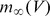 of the WB model instead of the Boltzmann kinetics, or when an exponent 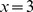 was used, which in the original Hodgkin-Huxley model leads to a delayed activation of sodium channels [25]. For the latter model, Fig. 4 shows the dependence of the AP dynamics on the coupling strength and the fraction of cooperative channels. The onset rapidness defined as the slope of the phase plot at 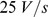, increases monotonically with increasing coupling strength. The AP onset is gradual when inter-channel coupling is weak (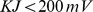, left inset in Fig. 4A), but becomes steep with 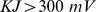. Notably, APs with steepest onsets were generated by models with a small fraction of strongly coupled channels (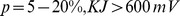, Fig. 4A). These APs were clearly biphasic, with a fast initial phase of cooperative activation followed by a slow rising phase of non-cooperative activation (Fig. 4A, inset bottom right). With increasing fraction of strongly coupled channels, the AP onset becomes less sharp (although still much faster than in models without cooperativity), and the two phases of the AP merge together (inset top right in Fig. 4A and Fig. 3D). In contrast, the peak rate of membrane potential rise increases monotonically with increases of both coupling strength and the fraction of cooperative channels.

APs of cortical neurons exhibit an onset rapidness of at least 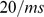 [10], [11], and are often biphasic [11], [27]. These features can be quantitatively reproduced in our model with a fraction 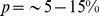 of tightly coupled (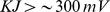) channels (Fig. 4). Assuming that each channel is coupled to 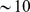 neighbors, this would imply that opening of a single channel leads to a 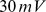 shift of the activation curve of its coupled neighbors. Such a strong inter-channel coupling is expected to lead to highly synchronized gating such that a cluster of coupled channels behaves as one functional unit. Exactly this type of highly synchronized opening and closing of channels was observed in the direct reports of coupled activation [2], [3], [5]–[8] (see however [28]).

In Hodgkin-Huxley type models, AP onset rapidness and threshold variability (standard deviation of the threshold, defined as voltage at which 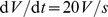) are intrinsically antagonistic [10]. This was not the case in the cooperative model. With strong channel cooperativity, APs are initiated at the discontinuous jump of 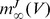 at voltage threshold 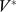:
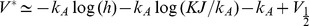
(11)This relation implies that the threshold variability caused by different levels of channel inactivation 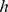 is not affected by the coupling strength 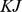. Numerical simulations of the model defined by Eqs. (1–4) subject to fluctuating input current confirmed this analytical result. With weak channel coupling, threshold variability and AP onset rapidness were antagonistic, but at critical coupling strength (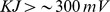) this relation was reversed, and APs with faster onsets expressed higher threshold variability (Fig. 4B,C). In neurons, variability of spike threshold may be further increased due to modulation of cell excitability by inhibition and potassium conductances [29], [30].

### Neuronal Encoding in a Model with Cooperative Channels

**Figure 5:**
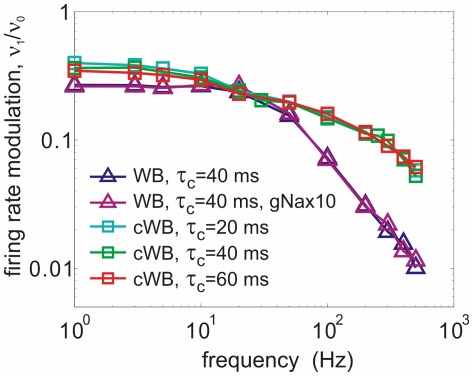
Cooperative channel gating improves the response of a conductance-based model to high-frequency inputs. Frequency response functions for the Wang-Buzsaki model with independent sodium channels and a standard or 10× fold increased density density of sodium channels (WB, triangles); for cooperative WB model with 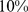 of cooperative 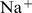 channels, 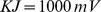, measured with 3 different correlation time constants of background noise (cWB, squares).

What are the coding properties of AP generators with cooperative channel gating? We assessed temporal properties of population coding by using the established approaches of measuring frequency response function [12]–[14], [16], [17]. Encoding of signal of a frequency 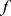 by a neuronal population is characterized by the modulation of the population firing rate 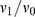 by the frequency 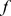. Plots of the firing rate modulation 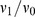 against the input signal frequency (Fig. 5) show that modulation by high frequency signals is improved substantially in the models with cooperative channels gating. At input frequencies 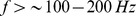, the modulation in these models was almost one order of magnitude larger than in the models with uncoupled channels. For high frequency signals, the modulation gain in models with cooperative channels decays roughly exponentially, deviating from power law behaviors reported for conventional conductance based models [12]–[17]. This difference was robust. It remained unaffected by changing time constants of the background noise (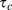) from 20 to 60 ms, or by a 10-fold increase of 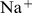 channel density in non-cooperative model that led to a strong increase of the peak 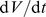 of APs (Fig. 5), but did not improve encoding of high frequencies. These results show that the presence of a small fraction of channels with cooperative gating can dramatically improve the encoding of high frequencies by populations of spiking neurons.

## Discussion

In summary, here we characterized a new class of models for AP generation with a small fraction of channels expressing cooperative gating. Our analysis demonstrates that these models robustly reproduce onset dynamics and waveforms of APs in neocortical neurons [10], [11], and predict an improved encoding of high frequency inputs - a recently described feature of neocortical population coding [19], [31], [32] that so far could not be captured by canonical conductance based models [14]–[17].

The AP waveforms of cortical neurons often exhibit two distinct components in the phase plot [11], [27]. In the past, the biphasic AP waveform in central neurons has been attributed solely to a lateral current invading the soma from the axon [11], [27], [33]. This lateral current was suggested to explain the fast onset dynamics and threshold variability of APs in the soma of neocortical neurons [11]. This scenario requires a strong current source in the proximal axon to depolarize the extended somatic membrane. However, direct evidence of an overhelmingly strong lateral current in neocortical neurons is missing. On the contrary, a recent examination of current density in the proximal axon of cortical pyramidal neurons strongly suggested that axonal current densities are much smaller than previously thought but on the same order as somatic current [34]. In addition, recent computational analyses showed that when APs are initiated in the axon initial segment about 30–50 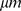 away from the soma, as has been demonstrated for neocortical neurons [35], [36], lateral currents alone are insufficient to explain fast onset of somatic APs [37]. Our results raise the possibility that the first AP phase reflects activation of a cooperative channel fraction. A likely scenario is that both lateral currents and a small fraction of cooperative channels contribute to the biphasic waveform and fast onset dynamics of somatic APs. Relative contribution of these two factors in shaping somatic APs awaits further investigation since their contributions are indistinguishable with available electrophysiological techniques.

One possible test suggested to distinguish between fast vs. slow AP onset dynamics at initiation site is to measure encoding properties of cortical neurons [20]. Prior theoretical analysis has demonstrated that coding abilities of spiking neurons critically depend on the onset dynamics of AP generators: model neurons with faster AP onset dynamics can encode higher frequency inputs than models with slow AP onsets [12]–[17]. It should be noted that prior analysis of spike encoding was performed using single-compartment models while analysis of encoding in models with realistic morphology is missing. Distribution of active and passive conductances over the dendritic tree and the axon of a neuron has a major influence on propagation and integration of postsynaptic potentials to the site of AP initiation [30], [38], [39]. However, it is logical to assume that spike encoding, i.e. transformation of membrane potential fluctuations at AP initiation site into spike trains, depends on properties of AP generators at the site of AP initiation, but do not depend on the location of an electrode that records spikes. If spike encoding is determined by AP onset dynamics at the site of its initiation, lateral current and the channel cooperativity hypotheses make distinct predictions on neuronal coding properties. Encoding of high frequencies should be enhanced in case of cooperative channel activation that produces rapid AP onsets at the initiation site, but not in the lateral current scenario, in which AP onset at the initiation site is slow. Recent studies have shown that cortical neurons can faithfully encode signal frequencies above 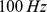, presented in a fluctuating background in slices in vitro [19], [31], [32] and in the whole brain in vivo [32]. The sensitivity of real neurons to the high frequency inputs is well above theoretically predicted encoding abilities of canonical conductance based AP generators, but is compatible with the high cut-off frequency of the model with a small fraction of strongly cooperative channels.

Channel cooperativity has been directly observed in a wide variety of biological ion channels, such as 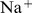 channels [2], 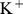, channels [3] 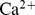 channels [5], [6] and neurotransmitter-gated channels [7], [8]. Our results show that waveforms and encoding properties of cortical APs are best reproduced in a model with a strong inter-channel coupling among a small population of cooperative channels. This is expected to lead to highly synchronized gating such that a cluster of coupled channels behaves as one functional unit. These requirements have two intriguing parallels in experimental data. First, the predicted behaviour perfectly agrees with existing experimental data on cooperative channels: almost all direct reports of coupled activation of ion channels have described exactly this type of highly synchronized channel opening and closing [2], [3], [5]–[8] (see however [28]). Second, neocortical neurons express sodium channels of several types, whereby 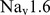 are concentrated at AP initiation site in the axon initial segment. These channels are tightly associated with submembrane anchoring proteins [40]–[42], and have a lower activation threshold than other sodium channels [43]–[45], and thus may represent plausible candidates for a fraction of cooperative channels. Although direct evidence for cooperative activation of 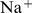 channels in axons of neocortical neurons is lacking, the ability of the model with cooperative channels to reproduce major observed properties of cortical APs, such as their rapid onset dynamics, threshold variability and ability to encode high-frequency signals, lends support to the cooperativity hypothesis [10]. Clarifying the molecular mechanisms of this type of gating will be crucial for understanding the computational capabilities of cortical neurons and networks.

## Supporting Information

File S1

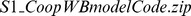
 **contains MatLab code for a single-compartment Wang-Buszaki model with a fraction 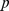 (default value 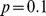) of cooperative sodium channels, with coupling strength** 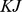 (**default value is** 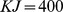). Model parameters other than cooperative channels are adapted from the Wang-Buszaki model [15], [24]. Zipped folder 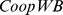 contains files 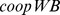, 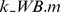 and 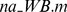 which should be in the same folder for the program to run. To run the model type in MatLab command window: 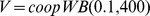. It will plot simulated membrane potential trace as a function of time and as phase plot, rate of membrane potential change against instantaneous value of the membrane potential.(ZIP)
